# Nurse Practitioner Knowledge, Attitudes, and Beliefs When Caring for Transgender People

**DOI:** 10.1089/trgh.2017.0048

**Published:** 2018-04-26

**Authors:** Catherine Paradiso, Robin M. Lally

**Affiliations:** ^1^ Department of Nursing, The College of Staten Island, The City University of New York, Staten Island, New York.; ^2^ College of Nursing, University of Nebraska Medical Center, Omaha, Nebraska.

**Keywords:** attitudes, beliefs, knowledge, nurse practitioners, transgender

## Abstract

**Purpose:** The aim of this study was to explore Nurse Practitioner (NP) knowledge, attitudes, and beliefs when working with transgender people and to inform about Practitioner education needs.

**Methods:** A qualitative descriptive design was used to explore (NP) experiences. Focused semistructured interviews were conducted in 2016 with 11 (*N*=11) NPs in the northeastern United States who represent various years of experience and encounters with transgender patients. The interviews explored NP knowledge attitudes and beliefs when caring for transgender patients and described their overall experiences in rendering care in the clinical setting. The interviews were professionally transcribed and analyzed independently and jointly by two investigators using conventional content analysis.

**Results:** Four main themes and six subthemes were identified: Main themes include personal and professional knowledge gaps, fear and uncertainty, caring with intention and pride, and creating an accepting environment.

**Conclusions:** NPs in this study perceive gaps in their knowledge that threaten their ability to deliver quality, patient-centered care to transgender patients, despite their best intentions. These findings have implications for changes in nursing practice, education, and research needed to address vital gaps in the healthcare of transgender people.

## Introduction

After years of discrimination in all areas of life, transgender people are now prominently included in the country's civil rights agenda. Healthcare discrimination is especially appalling. The National Transgender Discrimination Survey (NTDS) identified denial of healthcare, issues with provider ignorance of transgender and gender nonconforming health needs in preventative medicine, routine and emergency care, and transgender-related services in 2011 and again in 2016.^1,2^ Such discrimination reduces access and deters transgender people from seeking and receiving quality healthcare.^1^

In 2011, the Institute of Medicine (IOM) addressed health needs of transgender persons in their document “The Health of Lesbian, Gay, Bisexual, Transgender People: Building a Foundation for Better Understanding” describing stigma, discrimination, and lack of provider knowledge and training as barriers to transgender healthcare leading to significant health disparities.^3^ The need for transgender health research, although included under the umbrella of lesbian, gay, bisexual, transgender, and queer (LGBTQ), is receiving more prominence in the public and in academia. Improving the health, safety, and well-being of LGBTQ individuals is a Healthy People 20/20 objective.^4^ Also, sexual and gender minorities were officially designated as a health disparity for National Institute of Health research in 2015, raising consciousness in the research community and making funding available.^5^ Transgender care should, then, be an education and research priority for nursing.

Transgender healthcare is currently not required in medical provider education.^6,7^ Gaps in medical curriculum leave providers (Physicians, Physicians Assistants, NPs) unaware of evidence based standards,^8,9^ making access to care a barrier to basic health services.^10^

Nurse Practitioners (NPs) are prepared with 2–3 years of graduate education. There is no curriculum requirement to specifically include transgender health, but rather address any transgender issues as diversity in general.^11^ Moreover, most general nursing education programs have not included transgender issues at all into their curriculum and spend a short amount of time on the topic, about 2 h.^12^ To the best of our knowledge, there is only one published article on integrating LGBTQ content into a NP program.^13^ NPs increasingly provide primary and specialty care for a variety of populations and could improve access to and quality of care for transgender patients. There are no published studies that have explored the attitudes, beliefs, or educational needs of NPs when providing transgender care.

## Background

### Lack of data

Attempts have been made to estimate the population of transgender persons in the United States. The Williams Institute has estimated that 0.6% of adults, about 1.4 million, identify as transgender in the United States. They provide the first state-level estimates of the percentage of adults who identify as transgender.^14^

Research on transgender health is scant due to limited epidemiologic data.^8,15^ Academic researchers agree that the lack of epidemiologic data and an absent standard lexicon of definitions obstruct research. Larger studies to acquire evidence-based prevention data and plan care for the transgender population are needed, including a dedicated, national research infrastructure. Nationally, studies are needed that identify health promotion needs of this special population, training needs of providers, and strategies to achieve safe effective care for transgender people at all staged of transition.^15,16^

### Complexity of needs

Transgender healthcare needs are complex. As individuals transition into their identified gender and as they move through life, they may seek care from specialty providers such as urology, surgery, or gynecology. In addition to transgender-related services, primary prevention, routine, and emergency care are needed by all people, so provider understanding of how to care for transgender people is always necessary in healthcare settings. Healthcare provider competence is especially important in transgender reproductive health because of unique needs. For example, health promotion includes cancer screening for retained birth organs. Another example is that of breast cancer risk. Bazzi et al., (2015) found transgender patients were less likely than cisgender patients to adhere to screening guidelines.^17^ Screening guidelines for transwomen who are exposed to extended hormone use is not yet determined, so screening must be emphasized.

### Barriers to care

Barriers to quality care include the following: (1) reluctance of transgender patients to disclose gender identity when receiving medical care, (2) insufficient numbers of competent providers to care for LGBTQ issues, (3) insurance and policy barriers, (4) lack of culturally appropriate prevention services, and (5) discrimination.^3,8,18^ The importance of a competent provider and access to healthcare includes a greater likelihood of a medical evaluation before starting hormone therapy, obtaining hormone therapies from a medical provider, and a greater adherence to risk-reduction behaviors.^19^ Educating providers and creating a welcoming environment to remove feelings of stigma and discrimination are recommended to reduce barriers to care; however, one study found that as few as 20% of providers in OB/GYN receive formal training in transgender care and do not know clinical requirements following gender reassignment or routine health maintenance.^7,20^ Another study found that 79% of providers studied had never considered that their patient may identify as LGBTQ. In that study, all healthcare providers, except for nurses, demonstrated low levels of tolerance and respect. Nurses demonstrated the highest levels of tolerance and respect for transgender people.^6^

Providers lack comfort caring for this population compared to caring for lesbian and bisexual patients, regardless of years of experience.^20^ For example, discomfort in communication during transgender health encounters has been identified by Lurie (2005) who found that physician providers desired to treat transgender patients respectfully but admitted discomfort and lack of tools for asking specific questions during assessments.^21^ One specific area of discomfort is in meeting the psychological support needs of transgender patients, especially when behavioral healthcare is necessary. Providers describe patients with many behavioral health needs, some of which they are not prepared to meet because of a lack of understanding.^22,23^ Transgender people describe anticipating that providers will not know how to meet their needs and therefore avoid medical encounters.^22^

### Education can remove barriers

Healthcare provider education can remove barriers for transgender individuals. Lelutiu-Weinberger et al. found improvement in licensed and unlicensed medical staffs' knowledge and attitudes and a more welcoming clinic physical environment after training.^19,24^ Exposure to transgender individuals, whether in person or through videotape training, increased confidence levels and established a more positive attitude and performance of more comprehensive physical examinations when compared to medical staff and students who had no exposure.^6,25^

Guided by this evidence, this study aimed to answer the following research questions: What are NPs' attitudes, beliefs, and level of knowledge regarding the care of transgender individuals? and What do NPs describe as current gaps in Advanced Practice education pertaining to the care of transgender individuals?

## Project Design

A qualitative descriptive design was used. Focused semistructured interviews about the NP experiences were conducted in 2016. Semistructured interviewing allowed subjects to express openly, deeply, and in detail their experiences and feelings, when working with transgender patients.^26^ This study was approved by the Primary Investigators's university Institutional Review Board.

### Sample/participants

Purposive sampling was used to identify NPs with maximum variation in their clinical encounters with transgender patients. Maximum variation allows exploration of similar and unique experience across a broad range of individuals and was thus deemed the best method to answer the research questions.^26,27^ Participants were recruited from clinical practices and Universities in the Northeastern United States through the lead author's faculty and clinical contacts informing colleagues about the study. Criteria for inclusion were that NPs must have cared for at least one transgender patient. Table 1 describes the sample demographics

**Table 1.** **Subject Demographics**

Subject	Years in nursing practice	Nurse practitioner licensure	Nurse practitioner years	Education	Estimate number of transgender patients	Recent care
1	31	Family NP	7	MSN	5	Currently
2	12	Adult NP	4	MSN	10	Currently
3	40	Women's Health NP	24	PhD	3	6 years ago
4	8	Adult NP	5	MSN	100	Currently
5	18	Adult NP	8	DNP	15	Currently
6	14	Psyche.MH NP	3	MSN	6+	Currently
7	35	Family NP	16	MSN	3	6 months
8	6	Family NP	2	MSN	2	1 year ago
9	30	Nurse Midwife	21	DNP	>10	Currently
10	30	Family NP	20	MSN	100	6 months
11	25	Women's Health NP	8	DNP	4	1 year ago

NP, nurse practitioner.

A final sample of 11 NPs participated in this study. After, it was believed that data saturation had been reached (e.g., subsequent interviews were not providing additional data). The lead author purposefully sought out NPs with similar and dissimilar experiences to the first seven participants to confirm and/or disconfirm the initial data,^22^ thus adding to the credibility of the findings.^26,30^

### Data collection

Data were collected over a 4 month period. Following informed consent, focused, semistructured interviews were conducted in person (*n*=5) or via video conferencing (*n*=6) and digitally recorded.

An interview guide was used to maintain consistency in initial open-ended questions. These questions were followed by probing questions to obtain detail about the experience. All interviews were conducted by the lead author who maintained a journal of thoughts immediately following each interview. Key words were highlighted in the journal for analysis. Interviews were professionally transcribed.

### Analysis

Conventional content analysis was chosen for analyses of these data since this method is best used when a study design seeks to describe experiences with limited existing theory and research and to provide knowledge and understanding of the phenomenon under study.^26^ Analysis was ongoing throughout data collection. The first author read each transcript thoroughly to acquire the essence of each interview, then reread each interview multiple times to derive codes that captured the key concepts. Notes were taken of first impressions associated with quotes that exemplified key concepts. As analysis progressed, themes were identified that reflected associated concepts. Coded data were continuously compared with new data and themes.^27^ The second author coded the interviews independently and then reviewed the codes, themes, and subthemes developed by the first author identifying similarities and differences. An ongoing discussion between the authors resolved differences and resulted in collapsing and expanding subthemes throughout the analysis and development of the final article.^28,29^

### Rigor

This work was conducted with attention to credibility and dependability of the study data.^30^ An audit trail of the transcripts, coding, and decisions on themes and subthemes was maintained. The lead author also maintained a reflective journal containing her impressions throughout data collection. Credibility of this work is supported by independent and joint coding and theme development by the two authors; one (second author) experienced in qualitative research method and acting as a method and analysis coach to the lead author. The lead author is a NP with 15 of years of experience and a nurse educator, for whom this research is her Doctor of Nursing Practice scholarly work. Her professional background provided the lens through which these data were interpreted. Additional processes to support credibility included constant comparison of developing coding and themes and selecting interviewees later in the data collection process who represented varied experiences and professional backgrounds whose data could challenge initial data. All interviews were conducted one-on-one by the lead author, who does not have experience with care of transgender patients.

Interviews were conducted in a location chosen by the NPs to support interviewee privacy and comfort in sharing opinions on this sensitive subject matter. Finally, rigor was supported through sharing the article with an experienced DNP practicing in transgender health, to obtain input of the congruence of the work with current practice as the article was finalized.

## Results

Four predominant themes and six subthemes were identified. Themes included knowledge gaps, uncertainty and fear, caring with intention and pride, and creating an accepting environment.

### Knowledge gaps

Personal and professional knowledge deficits were described by all NPs, as experienced by themselves and their colleagues. NPs described transgender individuals' needs as very complex, involving behavioral health, gender, and transition care needs superimposed upon the usual care required by all people. Opportunities to provide care for transgender patients both highlighted NP knowledge deficit and provided chances to learn from their patients as well.

### Personal knowledge gaps

#### Patients have to teach providers

NPs' personal gaps in knowledge, resulting from a lack of resources and a minimal evidence base to guide practice, caused patients to have to teach their NPs about transgender care. Teaching from patients included making NPs aware that they still retained their birth organs, or that hormones may increase health risks of certain conditions. An NP described an example of her encounter with a female patient who informed the NP that she had a penis “I said to her ‘Would you be willing to educate me’ because better I should learn from a patient than reading a book.” (Subject #2). An experienced NP shared that learning from patients is ongoing and enhanced by asking questions.

“So stating to the patient, ‘if I misstep and I misspeak and I refer to you as something that makes you uncomfortable, if I say something or ask you something that makes you uncomfortable, it's not my intention to do that, but please stop me and correct me.” (Subject #9)

#### Lacking resources

Knowledge of transgender care had to be acquired, but NPs experienced frustration over the lack of available published evidence about transgender care. One NP described her efforts, including turning to the media for information, “I did some reading …, but there wasn't a lot to read. It was only after meeting transgender people like that I ever did anything to read up on it and try to watch it on TV if there was something” (Subject #3).

NPs also did not know where to obtain knowledge on terminology to support their communication with transgender patients. These nurses found that variations in terminology for describing individuals and anatomical changes exist within the transgender community, but are not necessarily known by providers. NPs described their dilemmas when even words that are automatic, such as “Mr.” or “Ms.,” may be incorrect or clinical requirements, such as cancer screenings protocols, are not clear for a transgender individual who may have internal organs of the opposite gender. NPs' insecurity with basic communication created awkwardness and caused them to be hesitant to speak and treat their transgender patients, despite the desire to provide quality care.

“I started self-teaching, what would help me would be to know a little bit more about the resources that are out there, because I don't even really know where my lapses of knowledge are. But every year I learn something new. I suppose I'm self-motivated because I care about the population.” (Subject #2)

### Professional gaps in knowledge

Regardless of how recent their education, all the NPs in this study expressed that transgender care had not been part of their graduate curriculum. The absence of education in transgender care was seen as a flaw. “There was nothing from the faculty. I would say that the training is minimal to nonexistent” (Subject #4).

Nursing faculty confirmed the perceptions of these NPs. A NP faculty member with many years of transgender care experience stated,

“I can tell you it's not something I teach in my curriculum. I could also tell from sitting on the board for the [NP exam] writing… We don't test on it. There's so much to teach that we don't teach them [NPs] about it [transgender care]. But there are certain webinars and education programs that you can tap into, if you can find them.” (Subject #5)

More experienced NPs describe the lack of transgender health education available through continuing education.

“I have not received any other training. There's no in-services or CE credits that are required by the places I've been employed. You have to do everything about infection control and other things every single year, but there's not much. There really is very limited promotion of the information of transgender treatment.” (Subject #6)

They further identify the need for efforts to provide continuing education to practicing NPs.

“I think it should be an automatic put in place, that maybe there is a speaker one night that's transgender. Maybe have a speaker the following week that is not just transgender–I know that's what we're talking about–but maybe have a gay or a lesbian couple or person come in and speak about some needs or feelings that they have that we're not Addressing.” (Subject #7)

### Uncertainty and fear

The complexity of transgender care coupled with NPs' knowledge deficits caused NPs to experience uncertainty and in some cases fear of making errors during clinical encounters. Knowledge gaps resulted in awkward encounters, which in some cases made the NP appear transphobic and ignorant. “I said, ‘really, there's a penis in that underwear? You're the most beautiful woman I've ever seen. What the heck is the story here?” (Subject #3).

Fear of making a mistake in clinical judgment, embarrassment and awkwardness from unknowing, and worry about making patients feel disrespected were described as objectifying.

“There were two others (I cared for) and they were both born females who were in their hearts and their heads really male. They looked feminine to me and I had to keep saying to myself, that's a he, you idiot; don't call it a she. That would be an insult; don't do that.” (Subject #3)

An experienced NP described the fear he observed in nurses around him,

“Some Nurse Practitioners are afraid and they're afraid because they don't know. Some of them don't understand; they can't wrap their heads around it; they don't conceptually understand it [transgender]. They don't understand how to treat them. They are afraid to treat them; they are afraid to misstep.” (Subject #5)

NPs' responses demonstrated acknowledgment of uncertainty, differing degrees of knowledge deficit, and levels of confidence in care provision associated with gender affirming hormone therapy. “Their medications are administered differently, and I've tried to research why.” (Subject #1). Another NP clarified the need to remember that transgender patients are the same as all people.

“They have the same healthcare needs that everyone else does and I think that is what we all forget. We all look at it like, oh, you must see this and you must see that, but they all have hypertension, they all have diabetes, they all have dyslipidemia. We still need to treat them as people. We still treat the diagnoses, the illnesses, and their disease processes. If he's a transgender male, he can still get sinusitis.” (Subject #5)

Reproductive care presents additional complexity in care and an especially sensitive topic that could create animosity between the patient and NP. Transgender patients may have two sets of anatomy, and an inexperienced NP may not realize all of the nuances with regard to genital structure and associated medical needs, for example, a trans male will have a cervix and require cancer screening, leading to uncertainty and fear of making a mistake or insulting the patient. These are extremely sensitive issues to all people, and in a transgender person the NP must understand these differences, the care required, and how to communicate this understanding. Without knowing, an NP could misgender a patient during the encounter, reducing trust and rendering the encounter nonproductive for the patient. Empowering a patient with knowledge, supporting them in their decisions, informing, and guiding are more likely to have a good outcome, but hard for an NP with limited experience and skill to achieve. Below is an example of an NP thinking he was doing so, but the patient did not accept the information, most likely because the NPs approach was authoritarian instead of collaborative.

“I said, ‘well, when was your last pelvic exam?’ He only slept with HIV positive men, orally, anally, and vaginally, so there was a lot of opportunity for counseling. I said, ‘you really do need to have a pelvic exam,’ he pretty much thought I was the worst person in the world because I told him that.” (Subject #5)

Another NP experienced in reproductive care describes uncertainty and fear over providing appropriate care.

“Not awkward because of their life choice, awkward because I am not sure I am doing the right thing and I want to do right by the patient… I just felt woefully inadequate… I do not know what I am supposed to be looking for, specifically or *per se*, for each of these clients. It's not that I felt uncomfortable personally. It was just more I felt inadequate as a healthcare provider. That was the daunting part of it for me.” (Subject #11)

### Caring with intention and pride

This theme illustrates that NPs worked to overcome their fears by putting extra effort into the intentional care of each transgender individual and filling their own knowledge gaps. By intentional, the NP is referring to “constant awareness” Over time, NPs experienced increased pride over their personal and professional growth. The following two subthemes reflect this further.

### Intentional care balances complexity

Knowledge gaps and patient complexity required NPs to take more time to think critically.

“There's always an awareness that this patient in front of me is transgender, versus if the person in front of me is gay, or black, or purple. I might not even think about it…. if it [the encounter] is transgender I will always remember. There's a difference. It's intentional in the way I have to interact with a transgender person.” (Subject #2)

Behavioral health comorbidities within the transgender population were also identified as an area requiring NPs to focus intentional care. “There's a lot of psych hospitalizations for this population; there's a lot of suicidal ideation and attempts.” (Subject #6); another said *“*…. higher levels of depression, higher levels of substance abuse in the population. Did I say domestic abuse?” (Subject #2). One NP described psychological issues experienced by transgender persons in more depth.

“Mental health effects that are related to facing a lifetime of discrimination, which for a lot of transgender people starts in childhood, so that's pretty deep and formative. Parental rejection, homelessness, or being cut off from the central family at some point, sometimes rejection from a partner, boyfriend, or girlfriend during transition or thereafter.” (Subject #4)

An experienced NP described high-level intentionality in care and gave an example of the care he provided to a patient who was a female transitioned to male. He advises NPs when delivering care, requiring this level of intention, to be humble and ask the patient what is not clear. He makes a point to the listener that as a clinician he must think careful of what anatomy is present, so that misgendering does not occur and the patient can be advised appropriately.

“Do not be afraid to ask your patient about what pronoun they want used. Consider the anatomy…. When I said that [you need to have a pelvic exam] to him, he was like, ‘of course that makes sense.’ [He understood that] of course……, In describing his thinking ‘*I would have to think about his anatomy, her anatomy, her male anatomy’.”* (Subject #5)

Describing the NPs thoughts as he went through them in his head shows the level of concentration and deliberate thinking to make sure that he did not misgender the patient when talking with him.

### Growing pride and confidence in care

Acceptance, nonjudgmental attitude, and self-education contributed to the NPs' pride in personal growth and confidence that allowed them to deliver quality care to their transgender patients.

When possible, filling knowledge gaps and gaining experience in caring for transgender patients boosted NPs' confidence, discussing important but very sensitive subjects with patients, as in this example from an NP, experienced in caring for transgender patients.

“So I'll say to my transgender females, I need to do a rectal exam because I need to do prostate. For my transgender males I'll say, ‘I do need you to see gynecology because I do need them to do a pelvic.’ So, it's important to lay those lines out, and they understand.” (Subject #5)

### Creating an accepting environment

NPs in this study felt compassion, acceptance, and a desire to show respect in caring for transgender patients. One mainstay of nursing is the keen awareness of how the environment sends loud messages, and how all nurses should work to assure that the environment sends messages that communicate acceptance, and respect to all patients. NPs recognized that provider offices and other places in the system may unintentionally communicate exclusion and offend transgender people.

### NPs must meet people where they are

All the NPs described their wish to see transgender patients treated the same as all people. “I think that we should be open and listen to patients and investigate certain things, so that we can help them through it.” (Subject #1) Another added, “I think that's the most important part…, that we need to be able to accept those patients and listen to them as they have some concerns.” (Subject #7) Meeting people where they are may be the goal, but at times NPs with limited experience may create the uncertainty described in preceding sections, and unintentionally be unable to meet people where they are by misgendering. One NP was enlightened by a patient's experience during transition, illustrating the patient's personal struggle, and the NPs not knowing where the patient was; so hard to meet these patients where they are:

“… a female who was transitioning to male and complaining about what the testosterone was doing to her … how she was feeling bossy and kind of, not nasty, ‘I feel very male, like not happy male,’ and she didn't know if she could because what she thought of as nice behavior to people wasn't coming up in her brain and in her behaviors, …. That was very mind opening and eye opening also. I never thought about it that way.” (Subject #3)

In this example, the patient identifies as male, but the NP is referring to the patient as female. While misgendering may have no ill intention, when done during an encounter creates stress for the patient. This is an example of how lack of experience and knowledge may impede good intentions.

### The environment sends messages

One mainstay of nursing is the keen awareness of how the environment sends loud messages, and how all nurses should work to assure messages sent by the environment communicate acceptance, and respect to all. NPs recognized that provider offices and other places in the system may unintentionally communicate exclusion and offend transgender people. “The forms, all the forms and the data that we enter do not give a choice; it's male or female, which is non-inclusive” (Subject #5).

An experienced NP gave practical advice on sending inclusionary messages in the clinical environment,

“…. look at something as simple as your office. Are you identifying your environment that you're inclusive to everybody, that you have two men, two women, a man and a woman, this, that and the other, as simple as the picture, as simple as the signage, as simple as education and looking at the forms. NIH puts it well. I think they have male, female, male to female and female to male on all the actual forms, or you could just leave it blank and let them identify themselves.” (Subject #5)

## Discussion

This study explored the knowledge, attitudes, and beliefs of eleven NPs with varying degrees of experience caring for transgender individuals. It revealed NPs' knowledge gaps that resulted in uncertainty and fear while rendering care, an overall caring attitude, knowing that the environment must be inclusive, and a belief that NPs can and wish to render quality care, but lack necessary tools. It also revealed lack of education and availability of resources related to transgender health. While some NPs had more knowledge than others, most lacked comprehensive knowledge and a full understanding of transgender health issues. For example, some NPs in this study were confused about anatomical changes, and several confuse gender identity with sexual orientation. All identified receiving no formal education or continuing education and lacking awareness about what resources are available for self-directed learning. All NPs found the profession lacking a meaningful body of knowledge or clinical experts readily accessible and available. They were unaware of existing work and protocols by The World Professional Association for Transgender Health (WPATH) and other groups. All NPs in this study described that personal and professional knowledge deficits affected patient care, and that improvements in graduate NP curriculum pertaining to transgender health are necessary, as are continuing education opportunities.

These results are consistent with and findings from other studies.^31^ A recent publication^22^ found uncertainty among physician providers, while another^32^ found uncertainty among RNs caring for transgender patients. These studies also revealed a lack of provider education and experience in providing respectful care to transgender people, which are barriers that perpetuate existing disparities. Also, in accord with these studies, was our study's finding that NPs were aware that patients felt discrimination in their previous experiences with providers and had a desire to be respectful in their practices.

NPs in this study were aware of the importance of accepting of a person's identity, feel compassion for transgender individuals' social plight, and possessed a desire to understand the complexities involved in these patients' care. It was noted that even with NP growing knowledge and experiences with transgender patients, their use of language, such as, “[sexual] preference” as opposed to “orientation” and referring to a transitioned person as an “it” revealed unconscious biases. Some identified awareness of their own potential to misgender or insult a patient. Subjects wanted to convey respect, felt no bias in their heart, but were aware that they could portray themselves otherwise. This emphasizes the serious need for training. According to the Association of American Medical Colleges, education of students can work to overcome these biases.^33^ Bias originates from assumptions that are not accurate and are often unconscious.^33^ While NPs in this study perceived themselves as unbiased, not knowing that anatomical changes or proper communication takes the form of bias. Finding these biases among NPs who volunteered for this study and who expressed interest in improving their knowledge and welcoming transgender patients leads to questions about biases held by NPs who may be even less aware and committed to transgender care.

Lack of knowledge required delivering care with more intention. The need for constant awareness of their deficiencies was described by most of the NPs and is present because they were aware that their lack of experience could result in offending the patient. Ultimately, the NPs described an environment in which transgender patients and healthcare providers are not always comfortable with each other. Patients sense a lack of provider competence, and providers experience discomfort with their own lack of knowledge. These findings are consistent with patient experiences identified by in the literature.^21–23^

This study offered the participants the opportunity to self-reflect on the care they render. In doing so, they identified that knowledge deficits and uncertainty inhibit shared decision-making and full actualization of NPs' potential. The partnership between NP and patient is fundamental to providing quality care and is weakened if the NP partner is not prepared to inform, educate, guide, and instill confidence. The partnership is only strong when the NP recognizes the autonomy of the patient, and the patient feels respected and empowered with information and support to make their own decisions. Even if the provider is knowledgeable, an authoritarian approach will always weaken the partnership.

Intention and desire to render inclusive care was a strength held by these providers, but without education and resources to develop clinical competence, uncertainty, and fear resulted. The NPs worked to create an accepting environment as best as possible during encounters. However, they all identified that more must be done to reduce invisibility of the transgender patient. For example, some NPs worked in environments that routinely cared for transgender patients, so their knowledge included the need to have inclusive signage, forms, pictures, and subordinate staff who are properly trained. Other NPs worked in settings where transgender patient encounters were rare, and thus there were no system efforts toward inclusion. Thus, there existed room for improvement.

NPs advocate for patients in systems where they work, as members of professional associations, and in communities where they live. Advocacy for transgender people can be strengthened by knowledge about and exposure to the population. Results of this study point to a potential need to assist operational leaders with resolving challenges, for example, are the nondiscrimination policies strong and sufficiently inclusive? Do we hold staff accountable for adhering to them? How can we include nonbinary gender identification in our Electronic Medical Records? How can we participate in training ancillary staff? How do we direct staff when identified gender or name and existing demographic forms do not match? What public policies need changes and how can NPs become involved? Based on this study's findings, addressing such questions holds the potential to positively impact the care of transgender people through increasing the knowledge and ease with which NPs engage with transgender patients.

This study is important, as it revealed the need for more education and research in this field. Some elements are very basic, such as knowing to acknowledge, apologize, and correct mistakes in communication when they occur. As NPs' practice scope expands and the openness of the transgender community increases, more NP interaction with transgender patients will result. Due to a sparse evidence base, little is known about how NPs perceive experiences with transgender patients and whether NPs are able to provide quality, patient-centered care for these individuals. It is important to know about NP practice with this population so that NPs can understand the transgender community, and provide clinically competent care without judgmental attitude. Also important are studies that investigate the transgender patients' experience with NP providers. Best practices for health promotion and shared decision-making strategies for this population should also be researched.

Inexperience and unawareness weaken the role that NPs play in the broader role of health promotion and advocacy. NPs should lead the work of developing national health promotion goals for transgender people. Comfort with the population and evidence makes robust health promotion and prevention plans possible.

Further implications of this study are not only for NPs but also for nursing education as a whole. Based on our findings, nursing curriculum should more thoroughly prepare NPs to care for the transgender population. Improvements in training for new NPs about to enter practice and in continuing education for practicing NPs is a necessity and must detail needs of transgender people specifically, not be simply addressed as diversity training for LGBTQ issues. Most important is for NPs to reach understanding and accepting of the many ways that people may identify gender, and how to engage in respectful communication to establish trust. Health risks of the transgender community and where NP can find the latest evidence-based resources should be part of each NP's lexicon. Education of all nurses presently in practice, administration, education, and research are essential to improve transgender care throughout nursing and is the only way to change the culture of healthcare now experienced by transgender people from a place where they feel disregarded to one where they are comfortable. Strategies to provide education that is more inclusive has been identified,^13,34^ but more is needed. NP programs need to include and test on best practices from protocols, such as WPATH, with the same approach as used with other clinical guidelines and assure that all teaching materials are gender inclusive.

## Limitations

One limitation is that subjects were recruited from the North East, with nine from NYC. Most subjects had significant amounts of experience, so it was assumed that their knowledge, attitudes, and beliefs had evolved over time and were influenced by past experiences or lack of experiences. Another limitation is that subjects might have been hesitant to disclose negative attitudes because of the public attention toward the community and because these attitudes are counter-instinctual and not acceptable for nurses.

## Conclusion

Discrimination toward transgender people continues to be widespread. Discrimination and bias in healthcare delivery, even if unintentional, contributes to and supports the disparity in care. Findings in this study show that despite a desire to provide care, lack of experience with and education about transgender healthcare limit NPs in their role, potentially causing them to be among the group of providers who unintentionally support existing disparities. There is a dearth of evidence in peer-reviewed nursing literature for NPs to use when caring for transgender people. Knowing what is important to patients, how to properly communicate, and knowing what health conditions people are most at risk for are basics in the provision of care for all people. Ultimately, nursing must begin to research transgender issues and teach all levels of nurses, through graduate nursing education on quality care for transgender people to eliminate current disparities in care.
